# Cross-sectional study on the seroprevalence, reinfection, and associated factors of *Chlamydia trachomatis* among female sex workers in Guangdong Province, China

**DOI:** 10.1186/s12879-025-10650-x

**Published:** 2025-03-13

**Authors:** Peizhen Zhao, Lei Xu, Yinna Huang, He Huang, Junhe Chen, Weiming Tang, Lijun Mo, Qingqing Xu, Shujie Huang, Cheng Wang, Heping Zheng, Bin Yang, Yaohua Xue

**Affiliations:** 1 Dermatology Hospital, Southern Medical University, 2 Rujing Rd, Yuexiu District, Guangzhou City, China; 2 Southern Medical University Institute for Global Health, Guangzhou City, China; 3 Guangzhou Key Laboratory for Sexually Transmitted Diseases Control, Guangzhou City, China; 4 Medical Center for Public Health of Puning, Puning City, China; 5 Yingde Center for Chronic Disease Control, Yingde City, China; 6 University of North Carolina Project-China, Guangzhou City, China

**Keywords:** *Chlamydia trachomatis*, Female sex workers, Seroprevalence, Nucleic acid amplification tes, IgG, Antibody level

## Abstract

**Background:**

Female sex workers (FSWs) are at high risk of chlamydia infection, yet the seroprevalence among FSWs in China remains unclear. This study aimed to determine the seroprevalence of *Chlamydia trachomatis* and associated factors among FSWs in Guangdong Province, China.

**Methods:**

A cross-sectional study was conducted among FSWs in two cities in Guangdong Province. Participants provided serum and urine samples. Nucleic acid amplification test (NAAT) was used to detect *Chlamydia trachomatis* (CT) and *Neisseria gonorrhoeae* (NG) in urine samples. Enzyme-linked immunosorbent assay (ELISA) was used to detect chlamydia IgG antibodies in serum samples. Seropositivity was defined by IgG-positive results. Current chlamydia infection was identified by a positive NAAT result, while prior infection was indicated by positive chlamydia IgG and negative NAAT results. Reinfection was defined by positive results for both NAAT and chlamydia IgG. Moreover, positive ELISA results were reclassified into two categories: DU/mL values ≥ 37.89 (the median) were classified as high-positive and DU/mL values > 11 to 37.89 as low-positive. Sociodemographic data, CT and NG testing, and paper questionnaires were collected through face-to-face interviews. Univariate and multivariable logistic regressions explored factors associated with current CT infection.

**Results:**

A total of 435 serum and urine samples were analyzed. The median age of the participants was 32.0 (IQR: 27.0–37.0) years. Among the participants, 326 were CT IgG positive, resulting in an overall seroprevalence of 74.9% (95% CI, 70.6–78.9). The current infection proportion determined by NAAT was 12.2% (53/435) (95% CI, 9.3–15.6), significantly lower than the IgG seroprevalence. Seroprevalence was higher among those over 39 years (88.6%) compared to those under 20 years (62.5%). High seroprevalence was observed among NAAT-negative participants (74.3%, 95% CI, 69.7–78.7). Single, divorced, or widowed individuals had higher seroprevalence (78.4%, 95% CI, 72.9–83.1) compared to married ones (69.5%, 95% CI, 61.9–76.3) (*P* < 0.05). Among seropositive participants, 42 were both NAAT and chlamydia IgG positive, indicating a reinfection proportion of 12.9% (95% CI, 9.4–17.0). Among reinfections, 81.0% had high seropositivity and 19.0% had low seropositivity. Reinfection prevalence was highest in those under 20 years (50%, 95% CI, 18.7–81.3) (*P* < 0.05).

**Conclusion:**

This study found a high prevalence of anti-chlamydia IgG among FSWs, including those who were NAAT-negative. Additionally, there was a high reinfection proportion among Chinese FSWs. CT serological assays are increasingly recognized as valuable epidemiological tools. Younger FSWs and those new to transactional sex may be at higher risk and should be prioritized for community-based prevention interventions to reduce the burden of CT transmission. Overall, CT serological assays are increasingly recognized as valuable tools for epidemiological surveillance and intervention.

**Clinical trial number:**

Not applicable.

## Background

*Chlamydia trachomatis* (CT) is a common sexually transmitted infection, there were an estimated 131 million new cases of chlamydia worldwide among adults aged 15–49 years in 2012 [1]. CT is particularly prevalent among young women, with 4% of sexually active females aged 14–24 years infected. Most genital CT infections in women are asymptomatic, but untreated infections can lead to upper reproductive tract scarring and serious complications such as pelvic inflammatory disease, ectopic pregnancy, and tubal factor infertility [2–4].

Female sex workers (FSWs) are highly vulnerable to CT due to their numerous transactional sex partners and frequent unprotected sexual activities, significantly contributing to the transmission of sexually transmitted infections [5–7]. In China, a meta-analysis found a 16.4% prevalence of CT among FSWs, rising to 20.8% in those working in lower-tier venues [8]. Another meta-analysis demonstrated that the pooled prevalence of CT infection was 19.5% by NAAT among FSWs in China [6].

CT surveillance in many countries primarily uses nucleic acid amplification tests (NAAT) to detect current infections [9, 10]. Serological assays, however, can provide a history of past CT exposure [11–13]. About two-thirds of infected individuals have detectable antibody levels within three months of infection, and approximately one-third of women with single notification of CT infection remain seropositive 3–10 years after the initial infection [13]. Some studies report that IgG antibodies can persist for over 12 years post-infection, reflecting cumulative exposure to CT [14]. A meta-analysis found an association between CT serology and fertility-related and adverse pregnancy outcomes in women, based on low- to moderate-quality evidence [15]. Therefore, CT serology may provide valuable insights into cumulative disease burden and historical exposure. However, due to technical and biological constraints, its utility in accurately estimating the true incidence of infection is limited. However, serology could serve as a complementary tool to NAATs in specific contexts, such as prevalence studies or understanding long-term population exposure. Previous studies in the United States, Finland, and China indicated high CT seroprevalence among sexually active women [13, 14, 16]. However, the seroprevalence among FSWs in China remains unclear. Investigating this is crucial for developing effective prevention and intervention strategies. This study aimed to determine the seroprevalence and associated factors of CT among FSWs in China.

## Methods

### Study sites

Between April 2022 and August 2022, a cross-sectional study was conducted in Yingde and Puning, Guangdong province, China, at venues where transactional sex occurs, utilizing the local capacity of the FSWs outreach program.

### Study participants

Each city was equipped with an FSWs outreach team comprising at least one public health professional and one medical staff member, both possessing extensive experience in FSWs outreach services. These teams consistently delivered critical public health interventions, including condom promotion, reproductive health education, and sexually transmitted infections (STI) counseling. Participants were deemed eligible if they met the following criteria: (1) female sex assigned at birth, (2) engaged in transactional sex at least once in the past year, (3) aged 18 years or older, (4) Chinese nationals, (5) willing to provide urine and blood samples and complete the survey, and (6) provide written informed consent. FSWs who provided erotic or sexual content without engaging in physical sexual activity with clients are excluded.

### Data collection and variables

#### Mesaures

Paper questionnaires were utilized for data collection. The questionnaire used in this study was adapted from those used in our previous studies [7, 17], with modifications based on consultations with STI experts, local FSWs outreach service personnel, and policymakers. Before full implementation, a pilot survey with 20 volunteer female sex workers was conducted to refine questionnaire items and evaluate the operational capacity of local FSWs outreach services.

Data were collected at FSWs’ working venues, where each face-to-face interview was conducted by a member of the outreach team. Eligible participants completed the questionnaires independently with support from outreach workers. For those unable to read, the outreach workers read the survey questions aloud and assisted with the responses. All survey responses were handled anonymously and kept confidential. Each participant was assigned a unique identifier, which was used to match the questionnaire responses with the corresponding urine and blood sample results.

#### Sociodemographic and behavioral characteristics

The sociodemographic data included age (years), ethnicity (Han/Non-Han), marital status (married/ single, divorced or widowed), household registration in relation to the sex work venue (local city/other city but same province/other provinces), monthly income (< 700/700–1200/>1200 USD), education attainment (junior high school or below/ senior high school/technical school/college degree or above), and length of time working in the current location (< 6 month /6–12 months/>12 months). The sexual behavioral variables included having a steady partner (boyfriend or husband), consistent condom use during transactional sex in the past month (never/sometimes/always), and a lifetime history of chlamydia testing (yes/no) and in the last 12 months (yes/no). The variables’ responses were grouped for analysis based primarily on the frequency of response categories. Specifically, categories with a small number of responses were combined to ensure sufficient statistical power and meaningful interpretation of the results. Consistent condom use was defined as the consistent use of condoms in any oral, vaginal, or anal transactional sex. Linkage to prevention and care as secured by the FSWs outreach team during this research, as per current sexual health recommendations.

#### Urine sample processing

Samples were self-collected from female sex workers, each providing a 10 mL urine sample. After collection, transferred it into cobas^®^ PCR Media tubes, mixed upside down 5 times. These samples were subsequently transported to the Dermatology Hospital of Southern Medical University for NAAT analysis to detect CT and NG. Transport of the urine samples adhered to controlled temperature conditions and stored at 4 °C until processing.

DNA extraction and detection for CT and NG in FSW urine samples were conducted using the Roche cobas^®^ 4800 CT/NG system (Roche Diagnostics, Mannheim, Germany). This automated diagnostic assay utilizes a workstation to isolate nucleic acids from clinical specimens and includes a real-time instrument for detecting CT and NG. Experimental procedures strictly adhered to the manufacturer’s instructions.

#### Serum sample processing

Following the interview, a 5 mL blood sample was collected from participants by qualified medical professionals for laboratory testing. The bloods were allowed to clot at room temperature for an hour and transported to local hospital. Serum was separated by centrifugation at 1500 RCF for 15 min at room temperature and then stored at -80 °C until it was shipped to the Dermatology Hospital of Southern Medical University by dry ice. The specimens were frozen at -80 °C prior to assay. Serum samples underwent ELISA assays (DRG Instruments GmbH, Germany) to detect CT IgG antibodies. Average absorbance values of patient samples and controls were recorded as OD and OD-CO, respectively. Relative absorbance values were standardized to DU/mL according to the manufacturer’s protocol.

#### Case definition

According to the manufacturer’s protocol, DU/mL values > 11 were considered positive, while DU/mL values < 9 were deemed negative. DU/mL values ≥ 9 to 11 were categorized as indeterminate, requiring retesting after two to four weeks; if the second test also yielded values within this range, it was interpreted as negative. Due to the inability to collect additional samples within the specified timeframe, DU/mL values ≥ 9 to 11 were classified as negative based on expert judgment. To investigate factors influencing CT seroprevalence, a secondary analysis was conducted. ELISA results were reclassified into three categories: DU/mL values ≥ 37.89 (the median) were classified as high-positive, DU/mL values > 11 to 37.89 as low-positive, and DU/mL values ≤ 11 as seronegative.

CT infection was categorized into three groups based on CT NAAT results: individuals with a positive NAAT result were classified as currently infected, those with a positive IgG antibody and negative NAAT result were classified as previously infected, and those with both positive CT IgG and positive urine CT NAAT were classified as experiencing reinfection.

### Statistical analysis

The study presented sociodemographic characteristics, sexual behaviors, and CT testing history using descriptive statistics. Exact Clopper–Pearson 95% confidence intervals were calculated for CT IgG prevalence via ELISA and CT pathogen prevalence via NAAT. Categorical variable distributions were evaluated using chi-square tests (including Pearson chi-square, continuity correction, and Fisher’s exact test). Univariate and multivariable logistic regression analyses were conducted to explore associations between sociodemographic and behavioral variables and CT seropositivity. The multivariable model was adjusted for age, ethnicity, marital status, household registration in relation to the sex work venue, monthly income, educational attainment, and duration of residence in the current location. The study separately assessed the prevalence of CT reinfection and prior CT infection through stratified analysis. Subgroup analyses examined the characteristics of FSWs with available CT serology data, categorized by IgG ELISA results. The multivariable model was adjusted for age, ethnicity, marital status, household registration in relation to the sex work venue, monthly income, educational attainment, and duration of residence in the current location. Statistical significance was set at *P* < 0.05, and all analyses were conducted using STATA software version 17.

## Results

### Sociodemographic characteristics and sexual behaviors

During the study, 511 FSWs were initially recruited, of whom 76 did not provide serum or urine samples, resulting in 435 samples being analyzed. The median age of the participants was 32.0 (IQR: 27.0–37.0) years. The majority (78.2%, 340/435) were aged 20–39 years, identified as Han Chinese (89.2%, 388/435), and reported a monthly income of less than US $1200 (90.1%, 392/435). About two-thirds were single (61.6%, 268/435) and had worked in Guangdong Province for less than six months (52.2%, 227/435). Most participants reported consistent condom use during transactional sex in the past month (65.5%, 285/435) and had a steady male partner (61.6%, 268/435) (Table 1).

**Table 1:** Seroprevalence of anti-CT IgG and frequency of positive CT-NAAT among female sex workers in Guangdong Province, China, 2022 (*N* = 435)

Characteristics	Total	CT IgG positive		*P*	Total	CT-NAAT positive		*P*
		Number	Frenquency,%(95%CI)			Number	Frenquency,%(95%CI)	
**Total**	435	326	74.9(70.6–78.9)		435	53	12.2(9.3–15.6)	
**Age (years)**				**0.015**				0.094
< 20	16	10	62.5(35.4–84.8)		16	5	31.3(11.0-58.7)	
20–29	140	101	72.1(63.9–79.4)		140	18	12.9(7.8–19.6)	
30–39	200	145	72.5(65.8–78.6)		200	20	10.0(6.2–15.0)	
>=40	79	70	88.6(79.5–94.7)		79	10	12.7(6.2–22.0)	
**Ethnicity**				0.063				0.283
Han	388	296	76.3(71.7–80.4)		388	45	11.6(8.6–15.2)	
Non-Han	47	30	63.8(48.5–77.3)		47	8	17.0(7.6–30.8)	
**Legal marital status**				**0.037**				0.424
Married	167	116	69.5(61.9–76.3)		167	23	13.8(8.9–19.9)	
Single, divorced or widowed	268	210	78.4(72.9–83.1)		268	30	11.2(7.7–15.6)	
**Household registration in relation to the sex work venue**				0.348				0.58
Local city	69	56	81.2(69.9–89.6)		69	11	15.9(8.2–26.7)	
Other cities in this province	120	86	71.7(62.7–79.5)		120	14	11.7(6.5–18.8)	
Other provinces	246	184	74.8(68.9–80.1)		246	28	11.4(7.7–16.0)	
**Monthly income (USD)**				0.376				0.412
< $700	215	166	77.2(71.0-82.6)		215	25	11.6(7.7–16.7)	
$700∼$1200	177	131	74.0(66.9–80.3)		177	25	14.1(9.4–20.1)	
>$1200	43	29	67.4(51.5–80.9)		43	3	7.0(1.5–19.1)	
**Education attainment**				0.203				0.741
Junior high school or below	297	225	75.8(70.5–80.5)		297	38	12.8(9.2–17.1)	
Senior High school or technical school	125	94	75.2(66.7–82.5)		125	13	10.4(5.7–17.1)	
College degree or above	13	7	53.8(25.1–80.8)		13	2	15.4(1.9–45.4)	
**Length of time working in current location**				**0.038**				0.245
< 6 months	227	180	79.3(73.4–84.4)		227	22	9.7(6.2–14.3)	
6∼12 months	91	68	74.7(64.5–83.3)		91	13	14.3(7.8–23.2)	
Over 1 year	117	78	66.7(57.4–75.1)		117	18	15.4(9.4–23.2)	
**Consistent condom uses with clients in the past month**				0.712				0.694
Yes	285	212	74.4(68.9–79.4)		285	36	12.6(9.0-17.1)	
No	150	114	76.0(68.4–82.6)		150	17	11.3(6.7–17.5)	
**Steady male partner (boyfriend or husband)**				**0.037**				0.844
Yes	167	116	69.5(61.9–76.3)		167	21	12.6(8.0-18.6)	
No	268	210	78.4(72.9–83.1)		268	32	11.9(8.3–16.4)	
**Ever have chlamydia testing**				0.113				0.549
Yes	96	66	68.8(58.5–77.8)		96	10	10.4(5.1–18.3)	
No	339	260	76.7(71.8–81.1)		339	43	12.7(9.3–16.7)	

### Seroprevalence of CT

In the study of 435 FSWs, 326 tested positive for CT IgG, yielding an overall IgG seroprevalence of 74.9% (95% CI, 70.6–78.9). Seroprevalence varied significantly with age, ranging from 62.5% in those under 20 years to 88.6% in those over 39 years. Additionally, single, divorced, or widowed individuals exhibited higher seroprevalence (78.4%, 95% CI, 72.9–83.1) compared to married individuals (69.5%, 95% CI, 61.9–76.3) (*P* < 0.05). Concerning behavioral factors, participants without a steady male partner had a higher seroprevalence (78.4%, 95% CI, 72.9–83.1) than those with steady male partners (*P* < 0.05). There is also a correlation between the seroprevalence of CT and the length of time working in the current location, the shorter the duration, the higher the infection rate (*P* < 0.05). (Table 1).

### Current infection detected by NAAT

Among the 435 participants, the current infection proportion determined by NAAT was 12.2% (95% CI, 9.3–15.6), significantly lower than the seroprevalence of anti-CT IgG (74.9%, 326/435). Notably, the highest NAAT-positive proportion (31.3%, [95% CI, 11.0-58.7]) was observed in participants under 20 years old, whereas the lowest NAAT-positive proportion was found in those over 39 years old, which contrasts with the seroprevalence findings (Table 1).

### High seropositivity and low seropositivity

The prevalence of high seropositivity was higher for single, divorced or widowed FSWs compared to married FSWs (*P* < 0.05). The prevalence of high seropositivity was higher for income below 700$ compared to income above 1200$(*P* < 0.05). Among the current CT infections, 19.0% were low seropositivity and 81.0% were high seropositivity(*P* < 0.001) (Table 2).

**Table 2:** Characteristics of FSWs categorized by low-positive and high-positive anti-CT IgG levels in Guangdong Province, China, 2022 (*N* = 326)

Total	Total, *n* (%)	Low- positive Sample, *n* (%)	High- positive Sample, *n* (%)	*P*
	326(74.9)	163(50.0)	163(50.0)	
**Age (years)**				0.605
< 20	10(3.1)	3(30.0)	7(70.0)	
20–29	101(31)	50(49.5)	51(50.5)	
30–39	145(44.5)	73(50.3)	72(49.7)	
>=40	70(21.5)	37(52.9)	33(47.1)	
**Ethnicity**				0.443
Han	296(90.8)	146(49.3)	150(50.7)	
Non-Han	30(9.2)	17(56.7)	13(43.3)	
**Legal marital status**				**0.021**
Married	116(35.6)	68(58.6)	48(41.4)	
Single, divorced or widowed	210(64.4)	95(45.2)	115(54.8)	
**Household registration in relation to the sex work venue**				0.118
Local city	56(17.2)	21(37.5)	35(62.5)	
Other cities in this province	86(26.4)	46(53.5)	40(46.5)	
Other provinces	184(56.4)	96(52.2)	88(47.8)	
**Monthly income (USD)**				**0.047**
< $700	166(50.9)	73(44.0)	93(56.0)	
$700∼$1200	131(40.2)	71(54.2)	60(45.8)	
>$1200	29(8.9)	19(65.5)	10(34.5)	
**Education attainment**				0.629
Junior high school or below	225(69.0)	108(48.0)	117(52.0)	
Senior High school or technical school	94(28.8)	51(54.3)	43(45.7)	
College degree or above	7(2.1)	4(57.1)	3(42.9)	
**Length of time working in current location**				0.276
< 6 months	180(55.2)	87(48.3)	93(51.7)	
6∼12 months	68(20.9)	31(45.6)	37(54.4)	
Over 1 year	78(23.9)	45(57.7)	33(42.3)	
**Consistent condom uses with clients in the past month**				0.642
Yes	212(65)	108(50.9)	104(49.1)	
No	114(35)	55(48.2)	59(51.8)	
**Steady male partner (boyfriend or husband)**				0.105
Yes	116(35.6)	65(56.0)	51(44.0)	
No	210(64.4)	98(46.7)	112(53.3)	
**Ever have chlamydia testing**				0.408
Yes	66(20.2)	30(45.5)	36(54.5)	
No	260(79.8)	133(51.2)	127(48.8)	
**The history of positive results among individuals who ever have chlamydia testing**				0.955
Yes	13(19.7)	6(46.2)	7(53.8)	
No	53(80.3)	24(45.3)	29(54.7)	
**Chlamydia NAAT positive**				**< 0.001**
Yes	42(12.9)	8(19.0)	34(81.0)	
No	284(87.1)	155(54.6)	129(45.4)	

### Prevalence of CT reinfection

Among the 326 anti-CT IgG seropositive individual, 42 tested positive for NAAT. These latter FSWs were classified as experiencing reinfection in this study. Consequently, the overall prevalence of CT reinfection was 12.9% (95% CI, 9.4–17.0). Remarkably, reinfection was notably higher at 50% (95% CI, 18.7–81.3) among participants under 20 years old, contrasting sharply with older age groups (*P* < 0.05) (Table 3).

**Table 3:** Prevalence of chlamydia reinfection among FSWs in Guangdong Province, China, 2022 (*N* = 326)

Characteristics	Total	Positives CT-NAAT and CT IgG		*P*
		Number	Frenquency,%(95%CI)	
**Total**	326	42	12.9(9.4–17.0)	
**Age (years)**				**0.004**
< 20	10	5	50.0(18.7–81.3)	
20–29	101	13	12.9(7.0–21.0)	
30–39	145	15	10.3(5.9–16.5)	
>=40	70	9	12.9(6.1–23.0)	
**Ethnicity**				0.565
Han	296	37	12.5(9.0-16.8)	
Non-Han	30	5	16.7(5.6–34.7)	
**Legal marital status**				0.985
Married	116	15	12.9(7.4–20.4)	
Single, divorced or widowed	210	27	12.9(8.6–18.2)	
**Household registration in relation to the sex work venue**				0.261
Local city	56	10	17.9(8.9–30.4)	
Other cities in this province	86	13	15.1(8.3–24.5)	
Other provinces	184	19	10.3(6.3–15.7)	
**Monthly income (USD)**				0.597
< $700	166	22	13.3(8.5–19.4)	
$700∼$1200	131	18	13.7(8.4–20.8)	
>$1200	29	2	6.9(0.8–22.8)	
**Education attainment**				0.743
Junior high school or below	225	31	13.8(9.6–19.0)	
Senior High school or technical school	94	10	10.6(5.2–18.7)	
College degree or above	7	1	14.3(0.4–57.9)	
**Length of time working in current location**				0.566
< 6 months	180	20	11.1(6.9–16.6)	
6∼12 months	68	10	14.7(7.3–25.4)	
Over 1 year	78	12	15.4(8.2–25.3)	
**Consistent condom uses with clients in the past month**				0.812
Yes	212	28	13.2(9.0-18.5)	
No	114	14	12.3(6.9–19.7)	
**Steady male partner (boyfriend or husband)**				0.744
Yes	116	14	12.1(6.8–19.4)	
No	210	28	13.3(9.0-18.7)	
**Ever have chlamydia testing**				0.838
Yes	66	9	13.6(6.4–24.3)	
No	260	33	12.7(8.9–17.4)	

### Prevalence of prior CT infection

Among the 382 participants who tested negative for CT NAAT, 284 were found positive for CT IgG. This resulted in an overall seroprevalence of prior CT infection of 74.3% (95% CI, 69.7–78.7) among the NAAT-negative participants. Notably, seroprevalence rose significantly from 45.5% (95% CI, 16.7–76.6) in those under 20 years to 88.4% (95% CI, 78.4–94.9) in those over 39 years (*P* < 0.05) (Table 4).

**Table 4:** Prevalence of prior chlamydia infection among FSWs in Guangdong Province, China, 2022 (*N* = 382)

Characteristics	Total	Negative CT-NAAT and Positive CT IgG		*P*
		Number	Frenquency,%(95%CI)	
**Total**	382	284	74.3(69.7–78.7)	
**Age (years)**				**0.005**
< 20	11	5	45.5(16.7–76.6)	
20–29	122	88	72.1(63.3–79.9)	
30–39	180	130	72.2(65.1–78.6)	
>=40	69	61	88.4(78.4–94.9)	
**Ethnicity**				0.122
Han	343	259	75.5(70.6–80.0)	
Non-Han	39	25	64.1(47.2–78.8)	
**Legal marital status**				0.143
Married	144	101	70.1(62.0-77.5)	
Single, divorced or widowed	238	183	76.9(71.0-82.1)	
**Household registration in relation to the sex work venue**				0.269
Local city	58	46	79.3(66.6–88.8)	
Other cities in this province	106	73	68.9(59.1–77.5)	
Other provinces	218	165	75.7(69.4–81.2)	
**Monthly income (USD)**				0.551
< $700	190	144	75.8(69.1–81.7)	
$700∼$1200	152	113	74.3(66.6–81.1)	
>$1200	40	27	67.5(50.9–81.4)	
**Education attainment**				0.312
Junior high school or below	259	194	74.9(69.2–80.1)	
Senior High school or technical school	112	84	75.0(65.9–82.7)	
College degree or above	11	6	54.5(23.4–83.3)	
**Length of time working in current location**				0.104
< 6 months	205	160	78.0(71.8–83.5)	
6∼12 months	78	58	74.4(63.2–83.6)	
Over 1 year	99	66	66.7(56.5–75.8)	
**Consistent condom uses with clients in the past month**				0.783
Yes	249	184	73.9(68.0-79.2)	
No	133	100	75.2(67.0-82.3)	
**Steady male partner (boyfriend or husband)**				0.115
Yes	146	102	69.9(61.7–77.2)	
No	236	182	77.1(71.2–82.3)	
**Ever have chlamydia testing**				0.052
Yes	86	57	66.3(55.3–76.1)	
No	296	227	76.7(71.4–81.4)	

### Factors associated with CT seroprevalence

After accounting for age, ethnicity, marital status, household registration in relation to the sex work venue, monthly income, educational attainment, and length of residence in the current area, multivariable logistic regression analysis identified age (adjusted odds ratio (a*OR*) = 6.03, 95% CI: 1.68–21.70) as the only statistically significant factor associated with CT seropositivity (Table 5).

**Table 5:** Factors associated with the detection of anti-CT IgG among FSWs in Guangdong Province, China, 2022 (*N* = 435)

Characteristic		Anti-CT IgG (*N* = 435)			
	*n*(%)	cOR (95%CI)	*p*	aOR (95% CI)*	*p*
Age (years)					
< 20	16(3.7)	*Ref*		*Ref*	
20–29	140(32.2)	1.55(0.53–4.56)	0.423	2.18(0.71–6.70)	0.176
30–39	200(46)	1.58(0.55–4.56)	0.396	2.11(0.70–6.38)	0.186
>=40	79(18.2)	4.67(1.37–15.92)	**0.014**	6.03(1.68–21.70)	**0.006**
**Ethnicity**					
Han	388(89.2)	*Ref*		*Ref*	
Non-Han	47(10.8)	0.55(0.29–1.04)	0.066	0.53(0.27–1.05)	0.067
**Legal marital status**					
Married	167(38.4)	*Ref*		*Ref*	
Single, Divorced or widowed	268(61.6)	1.59(1.03–2.47)	**0.038**	1.45(0.88–2.38)	0.141
**Household registration in relation to the sex work venue**					
Local city	69(15.9)	*Ref*		*Ref*	
Other cities in this province	120(27.6)	0.59(0.29–1.21)	0.149	0.61(0.29–1.31)	0.203
Other provinces	246(56.6)	0.69(0.35–1.34)	0.275	0.81(0.38–1.70)	0.573
**Monthly income (USD)**					
< $700	215(49.4)	*Ref*		*Ref*	
$700∼$1200	177(40.7)	0.84(0.53–1.34)	0.462	1.07(0.62–1.84)	0.804
>$1200	43(9.9)	0.61(0.3–1.25)	0.176	0.95(0.41–2.20)	0.897
**Education attainment**					
Junior high school or below	297(62.3)	*Ref*		*Ref*	
Senior High school or technical school	125(28.7)	0.97(0.60–1.58)	0.903	0.94(0.55–1.61)	0.830
College degree or above	13(3.0)	0.37(0.12–1.15)	0.085	0.51(0.14–1.78)	0.289
**Length of time working in current location**					
< 6 months	227(52.2)	*Ref*			
6∼12 months	91(20.9)	0.77(0.44–1.37)	0.375	0.88(0.48–1.61)	0.672
Over 1 year	117(26.9)	0.52(0.32–0.86)	**0.011**	0.60(0.34–1.05)	0.075
**Consistent condom uses with clients in the past month**					
Yes	285(65.5)	*Ref*		*Ref*	
No	150(34.5)	1.09(0.69–1.73)	0.712	1.21(0.74–1.98)	0.447
**Steady male partner (boyfriend or husband)**					
Yes	167(38.4)	*Ref*		*Ref*	
No	268(61.6)	1.59(1.03–2.47)	**0.038**	1.25(0.74–2.10)	0.403
**Ever have chlamydia testing**					
Yes	96(22.1)	*Ref*		*Ref*	
No	339(77.9)	1.50(0.91–2.47)	0.114	1.21(0.70–2.08)	0.499

* Adjusted for age, ethnicity, marital status, household registration in relation to the sex work venue, monthly income, educational attainment, and length of residence in the current area

## Discussion

FSWs are highly susceptible to CT infection globally. The study uncovers a high seroprevalence of CT IgG and a significant reinfection proportion among FSWs in Guangdong Province, China. Our study provides valuable insights into the prevalence and associated factors of anti-CT IgG among Chinese FSWs, with important implications for future CT interventions in this population.

We found a notably high CT IgG seroprevalence (73.9%) among Chinese FSWs, exceeding proportions reported in other regions of China (54.7%), Finland (65.5%), the United States (30.0%), and Kenya(19.0%) [13, 16, 18, 19]. This proportion is comparable to studies conducted in Cambodia(81.7%) and Brazil(100.0%) [20, 21]. The elevated seroprevalence among FSWs is likely due to their prolonged involvement in transactional sex work and frequent sexual encounters with multiple clients. Our study identified age as a significant factor associated with CT seropositivity, showing a clear trend of increasing seroprevalence with age. This finding is consistent with previous studies in the United States(30.0%) and Haiti(41.7%) [16, 22]. FSWs, being highly sexually active, are more prone to CT infection, which stimulates the host immune response and antibody production. Serum antibodies serve as crucial markers of immunity following CT infection, indicating past infection. Previous research has shown that approximately one-third of individuals remain seropositive for CT-specific antibodies up to 3–10 years post-infection, with persistence strongly influenced by initial antibody levels and recurrent infections [13]. CT serology offers a valuable method for investigating the incidence of CT infection and understanding its epidemiology and prevention. While most studies use NAAT to detect active CT infection [9, 10, 17], serological assays can determine the prevalence of past infections [12]. Serological assays for CT have been used in several countries to assess epidemiology, quantify unmet needs, inform service planning, evaluate interventions such as screening and treatment, and assess new vaccine candidates [4, 19, 23, 24]. Given these findings, incorporating serological assays into FSWs sentinel surveillance programs through social assistance or policy support could provide more comprehensive data for CT control and prevention.

Our study identified a 12.8% reinfection proportion among Chinese FSWs, which is significantly higher than the proportions reported for reproductive-aged women in the United States (6.1%) and the general population in Northern China [2, 14]. One study suggests that while previous CT infections may provide some degree of protective immunity against reinfection, this immunity is partial and insufficient to prevent new infections fully [25]. FSWs are particularly vulnerable to CT reinfection due to frequent transactional sex activities, inconsistent condom use with private partners, and multiple sexual partners [26]. In our study, we also observed that younger FSWs had a higher risk of CT reinfection. This suggests that age may be a significant factor contributing to the increased susceptibility to reinfection, alongside the factors previously mentioned. Reinfection can lead to severe outcomes in women, including pelvic inflammatory disease, ectopic pregnancy, and infertility. Notably, half of the FSWs under 20 years old in our study experienced reinfection, highlighting the urgent need for targeted CT prevention measures for young women engaging in high-risk behaviors. Doxycycline post-exposure prophylaxis (doxyPEP) has been shown to significantly reduce the risk of acquiring certain bacterial STIs in populations at increased risk, making it a promising intervention for highly exposed groups such as FSWs [27]. Our study also found that only about one-fifth of FSWs had ever undergone CT testing in the lifetime, a proportion much lower than the proportion of FSWs tested within 12 months in German (65.4%) [28]. Test and treat, including FSWs clients, is a proven strategy for preventing early detection and onward transmission. Many developed countries, including the United States, England, and Australia, have implemented CT screening guidelines for sexually active women to increase testing rates [29–31]. However, China currently lacks such guidelines. There is an urgent need to develop appropriate screening strategies to increase chlamydia testing rates among FSWs in China. Evidence-based interventions, such as promoting health education, improving condom use skills, enhancing communication and negotiation abilities, providing outreach services, and increasing basic knowledge of sexually transmitted diseases, have been shown to significantly reduce the risk of CT reinfection. Additionally, innovative approaches, including pay-it-forward programs and pre-exposure prevention strategies, can further complement traditional methods to enhance their effectiveness in reducing reinfection risks [32].

In this study, we found that women with CT reinfection exhibited higher antibody levels, with 81.0% of reinfected FSWs showing high seropositivity. Previous research indicated that a fourfold increase in chlamydia antibody titers between assessments was used as the case definition for reinfection in IgG-positive subjects, suggesting that reinfection may lead to elevated antibody titers [14]. Similar findings indicated that reinfected women with high seropositivity were approximately three times more prevalent than those with low seropositivity [2]. Additionally, a linear association between CT antibody levels and self-reported infertility in women was established [33], with infertile women showing high seropositivity almost twice as often as those with low seropositivity [2]. A systematic review have linked CT antibody levels to tubal disease [15, 34]. High chlamydia antibody titers may therefore indicate an elevated risk of reproductive disorders [35]. FSWs with high seropositivity should receive increased attention due to their elevated risk of ectopic pregnancy and tubal factor infertility. Comprehensive reproductive health education, increased screening, interventions aimed at reducing the frequency of intercourse or the number of sexual partners, strategies to increase condom use, and prompt treatment of pelvic inflammatory disease are all crucial measures to mitigate serious reproductive health consequences [32].

This study has several limitations. First, the FSW participants were from two cities in Guangdong Province, which are covered by sexual and reproductive health services offered in their work settings, limiting both sample size and potentially reducing the generalizability of the findings. Second, it was challenging for FSWs with unclear results to return for follow-up blood tests after two to four weeks, leading us to classify DU/mL values in gray areas as negative. This may have influenced the positivity rate of CT seroprevalence. Third, to identify factors associated with CT seroprevalence, we used the median value to define high and low positive levels based on expert opinion, which requires further research to refine. Fourth, as a cross-sectional study, longitudinal serological research is needed to investigate the frequency and antibody dynamics of CT infection in FSWs. Fifth, the absence of CT testing on oral and anal mucosa may have resulted in an underestimation of current CT infection cases. Sixth, the questionnaire did not capture data on antibiotic use for CT prevention, epidemiological treatment, or other health conditions requiring antibiotics. This omission may have hindered the differentiation between actual CT infections and the effects of antibiotic usage, introducing potential bias in interpreting infection prevalence and treatment patterns. Seventh, the absence of data on non-participants in this study precludes a thorough assessment of potential selection bias. Drawing from existing literature and insights from the FSWs outreach team, we hypothesize that non-participants may have exhibited lower uptake of CT testing, limited awareness of CT infection, and a reduced perceived risk of contracting CT, factors which likely contributed to their decision not to engage in the study [7, 36].

## Conclusion

In conclusion, our study found a high prevalence of anti-*Chlamydia* IgG among FSWs, including those who tested negative for CT NAAT. We observed a high reinfection proportion among Chinese FSWs and demonstrated that seroprevalence increases with age. The use of CT serological assays as an epidemiological tool is gaining interest. Local community transmission prevention interventions prioritize younger FSWs and those who are new to transactional sex, as these groups may be at higher risk of infection and reinfection. Targeted efforts in these populations could effectively reduce the disease burden and contribute to broader public health goals. Overall, these findings further underscore the growing recognition of CT serological assays as indispensable tools for epidemiological surveillance and intervention.

## Data Availability

The dataset used in the study are available from the corresponding author on reasonable request.

## Abbreviations

FSWs: Female sex workers
NAAT: Nucleic acid amplification test
CT: Chlamydia trachomatis
NG: Neisseria gonorrhoeae
ELISA: Enzyme-linked immunosorbent assay
STI: Sexually transmitted infections
aOR: Adjusted odds ratio
doxyPEP: Doxycycline post-exposure prophylaxis
